# Novel Wavelet-Based Segmentation of Prostate CBCT Images with Implanted Calypso Transponders

**DOI:** 10.4236/ijmpcero.2017.63030

**Published:** 2017-08

**Authors:** Yingxia Liu, Ziad Saleh, Yulin Song, Maria Chan, Xiang Li, Chengyu Shi, Xin Qian, Xiaoli Tang

**Affiliations:** 1 Shandong Communication and Media College, Jinan, China; 2 Medical Physics Department, Memorial Sloan Kettering Cancer Center, New York, NY, USA; 3 Radiation Oncology, North Shore Long Island Jewish Health System, New Hyde Park, NY, USA

**Keywords:** CBCT, Prostate Segmentation, Wavelets, MWDH

## Abstract

Segmentation of prostate Cone Beam CT (CBCT) images is an essential step towards real-time adaptive radiotherapy (ART). It is challenging for Calypso patients, as more artifacts generated by the beacon transponders are present on the images. We herein propose a novel wavelet-based segmentation algorithm for rectum, bladder, and prostate of CBCT images with implanted Calypso transponders. For a given CBCT, a Moving Window-Based Double Haar (MWDH) transformation is applied first to obtain the wavelet coefficients. Based on a user defined point in the object of interest, a cluster algorithm based adaptive thresholding is applied to the low frequency components of the wavelet coefficients, and a Lee filter theory based adaptive thresholding is applied on the high frequency components. For the next step, the wavelet reconstruction is applied to the thresholded wavelet coefficients. A binary (segmented) image of the object of interest is therefore obtained. 5 hypofractionated Calypso prostate patients with daily CBCT were studied. DICE, Sensitivity, Inclusiveness and ΔV were used to evaluate the segmentation result.

## 1. Introduction

One important step of the Adaptive Radiation Therapy (ART) is the segmentation of the CBCT images—a step required for the adaptive planning. This is specially important for hypofractionated treatments. Many studies have been published on automatic segmentation of prostate CBCT [1]–[9]. Accurate segmentation of CBCT is challenging due to the daily variations in rectal and bladder fillings as well as the increased noise levels in CBCT images. In our institution, some hypofractionated prostate patients are treated by Calypso tracking system (Varian, Palo Alto, CA). Three beacon transponders are implanted to the prostate, so that the target motion during the treatment delivery can be tracked in real time. However, the metal transponders introduce artifacts to the CBCT imaging, which makes segmentation more challenging. Based on our knowledge, no study was published yet on the segmentation of prostate CBCT with implanted Calypso transponders.

We propose to segment prostate and surrounding structures in the wavelet domain. The major advantage of wavelets is the ability to perform local analysis, *i.e.* trends, breakdown points, discontinuities, etc. The Double Haar wavelet transform can make the image edge detection more effective. The moving window implementation can protect the details and smooth the noise [10]. Therefore, we use a combination of these two—the Moving window-based Double Haar (MWDH) transformation for our prostate segmentation.

Adapted thresholds are assigned to different frequency components after MWDH. In low frequency component, cluster algorithm is employed to obtain a threshold *T_L_* to classify the region of interest. In high frequency components, Lee filter theory is used to calculate the adaptive threshold *T_H_* after de-noising. The segmented result is obtained by wavelet reconstruction of the thresholded components. Physician contoured the structures, and these served as ground truth.

The rest of this paper is organized as follows: in section 2, we will present the wavelet based segmentation algorithm in more details. The experimental results are shown in section3 and followed by discussion. The last section will conclude this study.

## 2. Materials and Methods

**Figure 1:**
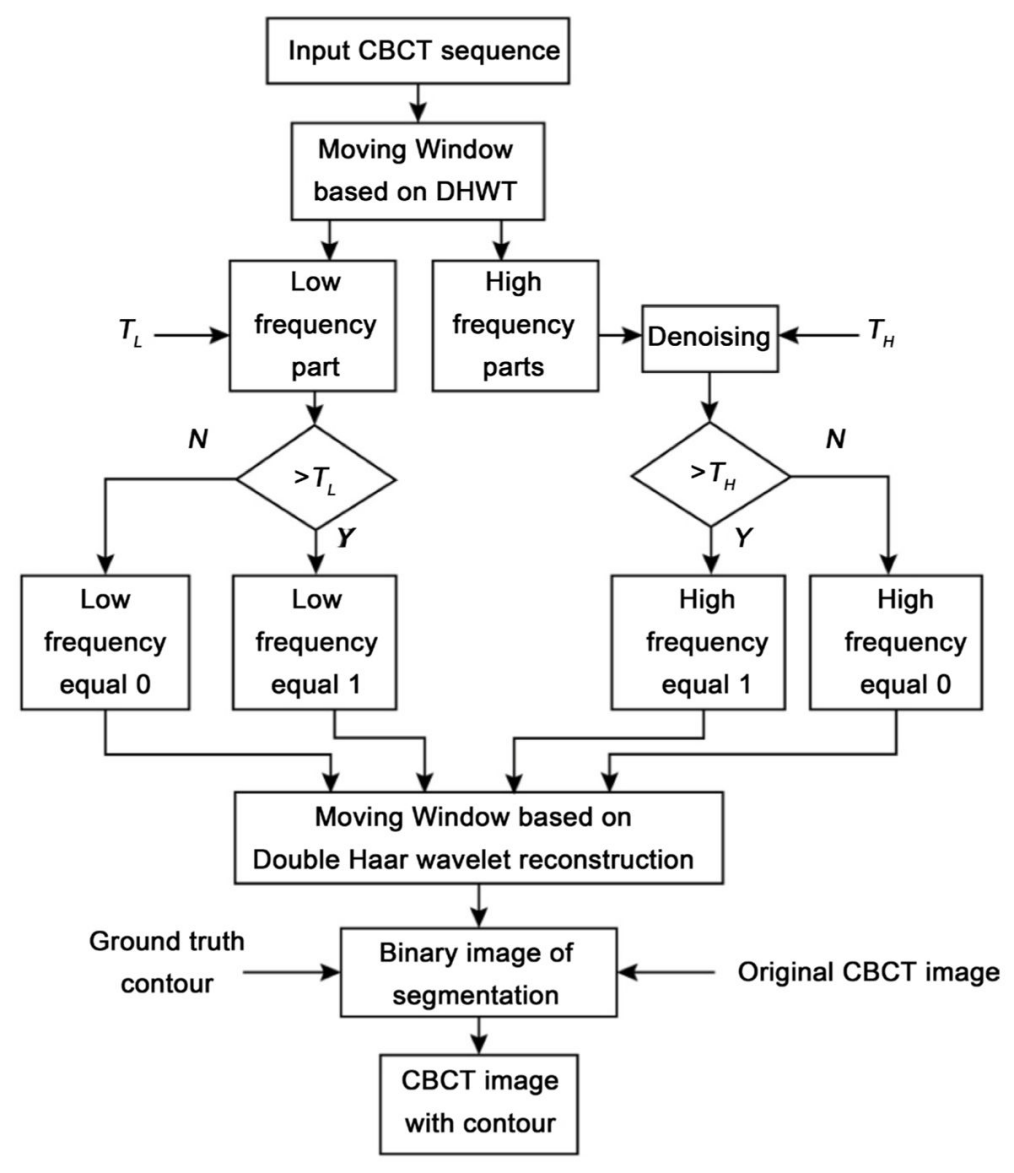
The flow chart of the segmentation algorithm based on MWDH. T_L_ and T_H_ are the thresholds for low and high frequency components.

The flow chart of the proposed algorithm is shown in Figure 1.

### 2.1. Moving Window Based Double Haar Wavelet Transform

**Figure 2:**
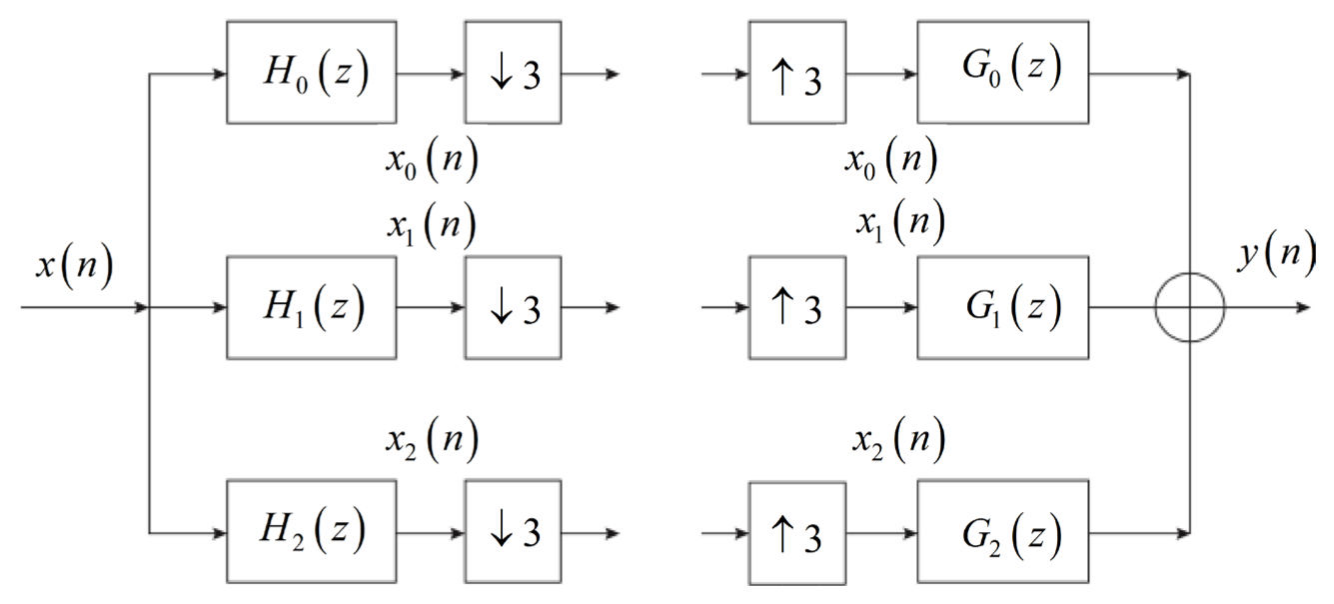
The structure of DHWT.

Different than the conventional two-channel wavelet transform, the Double Haar wavelet transform (DHWT) has three channels. As shown in Figure 2, the input signal *x*(*n*) is filtered by one low pass filter *H*_0_(*z*) and two high pass filters *H*_1_(*z*) and *H*_2_(*z*)*. x*_0_(*n*) (low frequency component/sub-band) and *x*_1_(*n*) and *x*_2_(*n*) (high frequency components/sub-bands) are the outputs. Then, sub-sampling is applied on each component to keep image size the same. For reconstruction, the interpolation needs to be applied first, followed by the reconstruction filters *G*_0_(*z*), *G*_1_(*z*), and *G*_2_(*z*).

### 2.2. The Adaptive Thresholding

In wavelet domain, low frequency components usually represent the main characteristics or identity of an image. The high frequency components, on another hand, are the nuance or details of an image. Considering these differences, different thresholding methods were applied on high and low components.

For low frequency component, the goal of thresholding is to group pixels of similar properties to a same group. Cluster algorithm [11] was designed to achieve this goal and was applied for low frequency component thresholding.

LEE filter can yield a local linear minimum mean-square error estimate of the original and give a better edge protective effect. Therefore, it was utilized for high frequency components thresholding.

For both cluster algorithm and LEE filter, image pixel statistics is needed to calculate the thresholding value. This is achieved by having user manually select a point in the structure of interest before the segmentation. Then a window is applied to collect the statistics.

### 2.3. Patient Information

Five hypofractionated prostate patients with prescription of 800 cGy × 3 and daily CBCT were studied. Each patient had 3 Calypso transponder beacons implanted, and the patients were setup and treated with Calypso tracking system. Two sets of CBCT image from each patient were studied. 3 × 3 moving window was used. The MWDH based segmentation algorithm was applied to segment and prostate, bladder and rectum.

### 2.4. Evaluation of the Segmentation

The structures were also contoured by trained expert, and these served as ground truth. We validate the proposed segmentation algorithm using the following metrics.

#### Dice Similarity Coefficient (DSC)

DSC measures the spatial overlap between two segmentations [12], and is defined as:

(1) $$\mathrm{DSC}=\frac{2(V_{\mathrm{seg}}\cap V_{\mathrm{ground}})}{V_{\mathrm{seg}}+V_{\mathrm{ground}}}$$

where *V_seg_* is the structure volume obtained by the proposed segmentation algorithm. *V_ground_* is the ground truth. DSC has a range of [0, 1], where 0 means no overlap, and 1 means complete overlap.

#### Sensitivity

The sensitivity reflects the probability that the automatic segmentation contour match the ground truth contour [12]. It is defined as:

(2) $$\mathrm{Sensitivi}ty=\frac{V_{\mathrm{seg}}\cap V_{\mathrm{ground}}}{V_{\mathrm{ground}}}$$

#### Inclusiveness (Incl)

The inclusiveness shows the inclusion of *V_seg_* within *V_ground_*, it reflects the probability that a pixel of the *V_seg_* also belongs to *V_ground_*. It is computed by:

(3) $$\mathrm{IncI}=\frac{V_{\mathrm{seg}}\cap V_{\mathrm{ground}}}{V_{\mathrm{seg}}}$$

Δ*V*: Δ*V* is the ratio of the difference between *V_seg_* and *V_ground_* over *V_ground_*, it is defined as:

(4) $$\Delta V=\frac{V_{\mathrm{seg}}-V_{\mathrm{ground}}}{V_{\mathrm{ground}}}\times 100\%$$

## 3. Results

**Figure 3:**
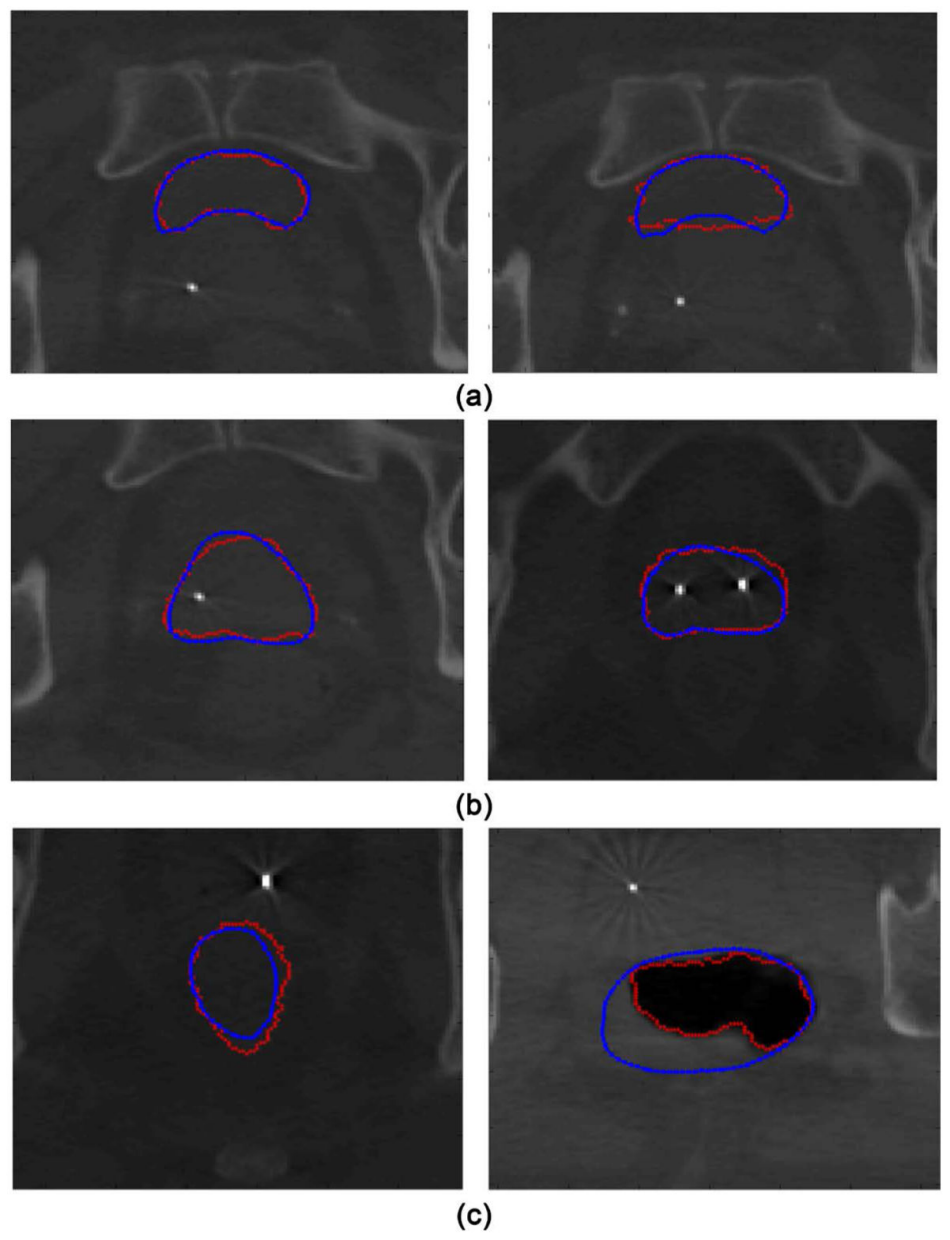
Contour comparison between the segmentation (red) and ground truth (blue). The ones with inferior segmentation results were plotted using thicker lines. (a) Bladder; (b) Prostate; (c) Rectum.

Table 1 lists the statistical results of the segmentation. Figure 3 displays examples of the segmentation results.

## 4. Discussion

Segmentation of prostate in important for adaptive radiation therapy [13]–[20]. One type of commonly used approach is deformable registration-based algorithm [4] [9]. Usually, a rigid registration is applied first to propagate the planning CT contours to the CBCT images. Then, iterative algorithm (*i.e.* Demons) is applied to calculate the pixel deformation flow/vector, until the given constrain is met. This works for some cases. However, when a large fraction of the propagated rectum and bladder contours were unacceptable, it provided a sub-optimal starting point for the deformable registration. This approach is difficult to generalize-the same measures and transformation constraints might not work for all the structures.

Another type is model/atlas based segmentation. As its name suggests, model needs to be built. The assumption of this approach is that structures of interest have a repetitive form of geometry. It involves expert manual segmentation of the structures of interest, and registration of the training examples to a common pose or model training to build the model [20] [21] [22]. This type involves significant amount of expert time. Expert contours usually involve human variations as well, which might be reflected in the built segmentation model.

Our proposed wavelet based prostate CBCT segmentation algorithm does not require deformable registration or model building. The moving window-based MWDH transfers the CBCT images to the wavelet domain, which contains high and low frequency components. We applied different thresholding technique to segment the different components. The final segmentation was achieved by the reconstruction of the thresholded wavelet coefficients. The algorithm is semi-automatic, as it requires user input to select a starting point. Our proposed algorithm achieved reasonable DICE index for all structures over all patients. However, it has challenges in two scenarios: 1) prostate with very low contrast; 2) rectum with significant amount of gas. The right hand side of Figure 3(b) illustrates the first scenario. The contrast of prostate was low, and the algorithm over-segmented the prostate. The right hand side of Figure 3(c) illustrates the second scenario, when the rectum was filled with significant amount of gas, the algorithm tended to segment the gas. We have tried to select the user point inside and outside of the gas, and did not observe noticeable improvement. Multiple user entered points might help these two kinds of situations, and we will include it in our future work.

Haar wavelet transform has many advantages. There is no need for multiplications. It requires only additions and therefore the computation time is short. Its input and output length are the same. Although the double Haar wavelet transform enhanced its ability to analyze the localized features of images, other more sophisticated wavelet transforms might analyze the high frequency components better and further improve the segmentation results.

## 5. Conclusion

The proposed algorithm appeared effective segmenting prostate CBCT images with the present of the Calypso artifacts under most common clinical scenarios. However, when the prostate contrast is low or there is significant amount of gas in the rectum, the algorithm might have inferior segmentation result.

## Figures and Tables

**Table 1:** Statistical results.

Structure	Metrics	Patient 1	Patient 2	Patient 3	Patient 4	Patient 5
Rectum	DICE	0.891	0.901	0.838	0.877	0.709
	Sensitivity	0.954	0.899	0.98	0.976	0.758
	Inclusiveness	0.836	0.846	0.734	0.797	0.983
	ΔV	15.53%	12.17%	34.30%	22.80%	27.27%
Prostate	DICE	0.773	0.818	0.811	0.87	0.888
	Sensitivity	0.926	0.886	0.933	0.928	0.938
	Inclusiveness	0.677	0.807	0.62	0.834	0.817
	ΔV	46.47%	36.90%	36.40%	23.30%	13.40%
Bladder	DICE	0.86	0.811	0.926	0.919	0.927
	Sensitivity	0.832	0.765	0.953	0.912	0.939
	Inclusiveness	0.912	0.92	0.899	0.931	0.919
	ΔV	14.73%	20.63%	10.13%	7.40%	2.95%
